# Largely Accelerated Arterial Aging in Rheumatoid Arthritis Is Associated With Inflammatory Activity and Smoking in the Early Stage of the Disease

**DOI:** 10.3389/fphar.2020.601344

**Published:** 2020-11-26

**Authors:** Nikolett Mong, Zoltan Tarjanyi, Laszlo Tothfalusi, Andrea Bartykowszki, Aniko Ilona Nagy, Anett Szekely, David Becker, Pal Maurovich-Horvat, Bela Merkely, Gyorgy Nagy

**Affiliations:** ^1^ Polyclinic of Hospitaller Brothers of St. John of God, Budapest, Hungary; ^2^ Heart and Vascular Center, Semmelweis University, Budapest, Hungary; ^3^ Department of Pharmacodynamics, Semmelweis University, Budapest, Hungary; ^4^ MTA-SE Cardiovascular Imaging Research Group, Heart and Vascular Center, Semmelweis University, Budapest, Hungary; ^5^ Department of Radiology, Medical Imaging Centre, Semmelweis University, Budapest, Hungary; ^6^ Department of Rheumatology and Clinical Immunology, Semmelweis University, Budapest, Hungary; ^7^ Department of Genetics, Cell- and Immunobiology, Semmelweis University, Budapest, Hungary

**Keywords:** rheumatoid arthritis, coronary calcium score, arterial aging, inflammatory activity, smoking-adverse effects

## Abstract

**Background:** Rheumatoid arthritis (RA) patients have a shorter life expectancy than the general population primarily due to cardiovascular comorbidities.

**Objectives:** To characterize arterial aging in RA.

**Patients and Methods:** Coronary calcium score (CCS) were available from 112 RA patients; out of these patients, follow-up CCS were measured for 54 randomly selected individuals. Control CCS were obtained from the MESA database (includes 6,000 < participants); arterial age was calculated from CCS.

**Results:** RA patients were significantly older (10.45 ± 18.45 years, *p* < 0.001) in terms of the arterial age than the age-, gender-, and race-matched controls. The proportion of RA patients who had zero CCS was significantly less (*p* < 0.01) than that of those in the MESA reference group. Each disease year contributed an extra 0.395 years (*p* < 0.01) on the top of the normal aging process. However, the rate of the accelerated aging is not uniform, in the first years of the disease it is apparently faster. Smoking (*p* < 0.05), previous cardiovascular events (*p* < 0.05), and high blood pressure (*p* < 0.05) had additional significant effect on the aging process. In the follow-up study, inflammatory disease activity (CRP > 5 mg/L, *p* < 0.05) especially in smokers and shorter than 10 years of disease duration (*p* = 0.05) had the largest impact.

**Conclusion:** Arterial aging is faster in RA patients than in control subjects, particularly in the first 10 years of the disease. Inflammation, previous cardiovascular events, and smoking are additional contributing factors to the intensified coronary atherosclerosis progression. These data support that optimal control of inflammation is essential to attenuate the cardiovascular risk in RA.

## Introduction

Rheumatoid arthritis (RA) is a heterogeneous autoimmune condition; it affects 0.5–1% of the population and is associated with disability and systemic complications (Turesson et al., 2006; Prete et al., 2011; Das and Padhan, 2017). Both genetic and environmental factors have a central role in the pathogenesis of the disease, and cigarette smoke is the strongest known environmental factor (McInnes and Schett, 2007; Baka et al., 2009; McInnes and Schett, 2011). In RA, ongoing inflammation leads to cartilage destruction, bone erosions, and subsequent joint deformities. Although the current treatment strategy, principally the widespread use of biological therapies, improved the outcome of the disease, the mortality rate is still considerably higher among patients with RA than among healthy persons and systemic complications, especially cardiovascular (CV) risk due to RA, and represent a significant challenge (Liu et al., 2017; Nagy et al., 2018). Although biologicals have a beneficial effect on the CV risk in RA, TNF and IL6 inhibitors often increase the total cholesterol and triglyceride levels (Roubille et al., 2015; Gabay et al., 2016; Giles et al., 2019). In addition to the traditional cardiovascular risk factors (hypertension, diabetes, smoking, hyperlipidemia, alcohol, and physical inactivity), the effect of chronic inflammation on cardiovascular mortality is a rapidly developing field of interest (Ormseth et al., 2015). Elevated CRP level is considered as a cardiovascular risk factor (Yousuf et al., 2013; Fonseca and Izar, 2016). It is noteworthy that the risk of acute myocardial infarction (AMI) in RA is similar to the risk of AMI in diabetes mellitus (Nurmohamed et al., 2015). Coronary artery disease in RA appears more often in multivessel form (Karpouzas et al., 2014). In RA, the inflammation is associated with the presence of high-risk plaques (Aubry et al., 2007; Karpouzas et al., 2014). Coronary calcium score (CCS) is a well-established diagnostic marker showing calcium deposits in coronary arteries. It is known to be influenced by several factors including age, gender, race (Greenland et al., 2018), smoking (Shaw et al., 2006), high CRP levels (defined as higher than 5 mg/L), cardiovascular disease, high blood pressure, and diabetes, although the connection between diabetes and CCS is controversial (Raggi et al., 2004; Giles et al., 2009). The CCS assessment is a noninvasive method that has a great value in cardiovascular risk stratification, showing a significant association with the medium- or long-term occurrence of major cardiovascular events (O’Rourke et al., 2000; Neves et al., 2017). The prevalence of coronary artery calcium (CAC) increases with age, ranging from 5% in a middle-aged cohort to more than 50% in an elderly cohort (McClelland et al., 2006). A meta-analysis including asymptomatic individuals indicated that those with coronary artery calcification above the median have an 8.7-fold increased risk of future coronary events (O’Malley et al., 2000). In addition, there are data indicating that progression in CCS is associated with higher risk of myocardial infarction (Raggi et al., 2000; Raggi et al., 2003), and coronary artery calcification adds information to the prediction of overall mortality (Shaw et al., 2003). It has been proposed that CAC can be used to estimate the arterial age in adults. Although the increased cardiovascular risk is widely accepted in RA, the risk factors associated with the chronic autoimmune disease are less clear. The traditional cardiovascular risk scores underestimate the real cardiovascular risk in RA (Crowson et al., 2012; Kawai et al., 2015; Wahlin et al., 2019). CCS is better in CV risk stratification in RA than in combinations of the traditional CV risk factors (Korley et al., 2017; Karpouzas et al., 2020). Here, we investigated the baseline and follow-up CCS of RA patients and studied its progression over time. Our present result underscores the impact of inflammation on the CV risk in RA, especially in the first ten years of the disease.

## Patients and Methods

### Patients and Controls

All RA patients were recruited in the rheumatology outpatient department of the Semmelweis University (Polyclinic of Hospitaller Brothers of St. John of God, Budapest, Hungary). Patients ≥ 18 years of age and diagnosed with RA (*n* = 112) according to the 2010 American College of Rheumatology/European League Against Rheumatism classification criteria (Aletaha et al., 2010) were enrolled. Exclusion criteria included concomitant autoimmune disease, except Sjögren’s syndrome, malignant diseases, chronic infections with or without fever, and known psychiatric disease. The demographic data and the clinical parameters of the patients are summarized in Tables 1, 2. Hypertension, diabetes mellitus, and hyperlipidemia were evaluated based on the standard criteria; smoking history was recorded (smoker/nonsmoker). Disease activity was evaluated by using the 28-joint counts and erythrocyte sedimentation rate–based (disease activity score/DAS28) score at each visit. Medications were recorded including glucocorticoids, NSAIDs, conventional and targeted disease-modifying antirheumatic drugs (DMARDs), and statins. Both national and institutional ethics committees approved the study, and informed consent was obtained from each individual [approval number: IF 567-4-2016]. This work was carried out in accordance with the Helsinki Declaration.

**TABLE 1:** Patient characteristics, categorical variables. dm: diabetes (Type I: 3, Type II: 14), bp_ high: high blood pressure, esr_high: for males: ESR > 15 if age is less than 50 years and 20 if age is above 50 years. For females: 20 if age < 50 years and 30 above 50 years. CRP above 5/mg/L.

Variable	No	Yes	Total	Percent %
Female	18	94	112	83.9
Smoking	61	49	110	44.55
DM	95	17	112	15.18
CVevent	95	17	112	15.18
HT	42	70	112	62.50
RF positivity	36	76	112	83.04
aCCP positivity	46	66	112	60.71
high ESR	87	25	112	22.32
CRP 5	71	41	112	36.61
Biological therapy	52	60	112	53.57

**TABLE 2:** Patient characteristics, continuous variables. The arteries are approximately 10 years older in RA than in the matched control group. Disdur: disease duration, CCS: coronary calcium score, CRP: C-reactive protein, artAge: calculated arterial age using Eq. 1, artAge_dif: arterial age difference from the median of the race-, sex-, and age-matched control population.

Variable	*N*	Mean	SD	Min	Max	Median
Age	112	63.00	11.40	35.00	84.00	64
Disdur	112	12.09	10.20	0.50	58.00	10
CCS	112	253.25	488.30	0.00	3,379.00	45
HDL	109	1.64	0.42	0.82	3.66	1.62
Chol	111	5.48	1.11	3.40	8.50	5.5
Hba1c	110	5.67	0.75	4.40	9.40	5.6
CRP	112	7.08	12.89	0.06	79.00	3.6
DAS	112	3.16	1.36	0.57	6.63	2.91
artAge	112	62.22	19.86	39.10	98.01	66.96
artAge_dif	112	10.45	18.53	35.35	52.56	6.35

Control population: the Multi-Ethnic Study of Atherosclerosis (MESA) database was used as control. MESA is a prospective cohort study with an aim to investigate predictors of cardiovascular risk factors; coronary artery scan was performed in 6,814 participants without apparent cardiovascular problems. We refer to this population as “healthy” population or “MESA” population (Blaha et al., 2016). Age-, gender-, and race-matched control data were generated by using the online CCS calculator (https://www.mesa-nhlbi.org/Calcium/input.aspx). Based on demographic data and the measured CCS, the online calculator provided the estimated probability of having higher than zero calcium, and the 25th, 50th (median), 75th, and 90th CCS percentiles of in a “healthy” (i.e., without apparent cardiovascular disease) population. Using these percentiles and inverse quantile transformation, we simulated 100 age-, gender-, and race-adjusted CCS for each patient in our study. In this way, the control population consisted of 100 age-, gender-, and race-matched subjects from the MESA database for each RA patient, altogether 11,200 subjects.

### Measurement of Coronary Calcium Score

All RA participants underwent non–contrast-enhanced, prospectively ECG-triggered scan of the heart using a 256‐slice multidetector CT (Brilliance iCT 256; Philips Healthcare, Best, The Netherlands) at the Heart and Vascular Center of Semmelweis University. Images were acquired in cranio-caudal direction during a single breath hold in inspiration, at 78% of the R‐R interval, with a slice thickness of 2.0 mm. The following acquisition parameters were used: 128 × 0.625 mm detector collimation, 270 ms gantry rotation time, 120 kV tube voltage, and 30 mA s tube current. The quantification of CAC was performed on the axial images on a per‐patient and per‐vessel basis using a semi-automatic software (Heartbeat-CS, Philips Healthcare, Best, The Netherlands). CCS were computed by the standard calcium scoring algorithm of Agatston (Agatston et al., 1990). Follow-up CCS measurement was performed for 54 patients.

### Determination of the Arterial Age and Arterial Age Difference (artAge_dif)

Arterial age is an easy to understand intuitive concept; it shows the apparent age of arteries using healthy population as a reference. Therefore, to help the clinical interpretation of the results, CCS were transformed into “arterial age” using the formula of McClelland et al. (2009):

arterial age=39.1+7.25⁡log(CCS+1). (1)

To facilitate the statistical inference, we introduced an additional variable called artAge_dif. The variable artAge_dif measures difference between the observed and the control arterial ages. For each patient, there were 100 age-, gender-, and race-matched controls, and we obtained artAge_dif by subtracting the median of the corresponding controls from each observed value.

### Statistical Analysis

Stata version 15 (StataCorp LP, College Station, TX) was used for statistical analysis and R (R Core Team, 2017) with several additional packages such as ggplot2 (Wickham, 2016) for additional programming tasks and visualization. The difference between the measured and MESA control population values was tested using the one-sample alternative of the tests; that is, we assumed that simulated control population is not different from the “true” population. Percentages of subjects having nonzero CCS in the observed and computer-predicted populations were compared with exact binomial test. The effect of factors that may influence CCS was studied by graphical analysis followed by univariate testing and multivariate linear regression modeling. Due to highly nonnormal distribution of the data, we gave preference to nonparametric methods such as rank-based tests or we took advantage of the “robust” option available for many procedures in Stata. To illuminate the trends, we fitted locally weighted polynomial regression commonly known as LOWESS. Descriptive summaries such as proportion, means, and standard deviation (SD) are provided for all clinically relevant baseline variables, and statistical significance level was set to *p* < 0.05, two-tailed.

## Results

### Accelerated Arterial Aging in Rheumatoid Arthritis

**FIGURE 1:**
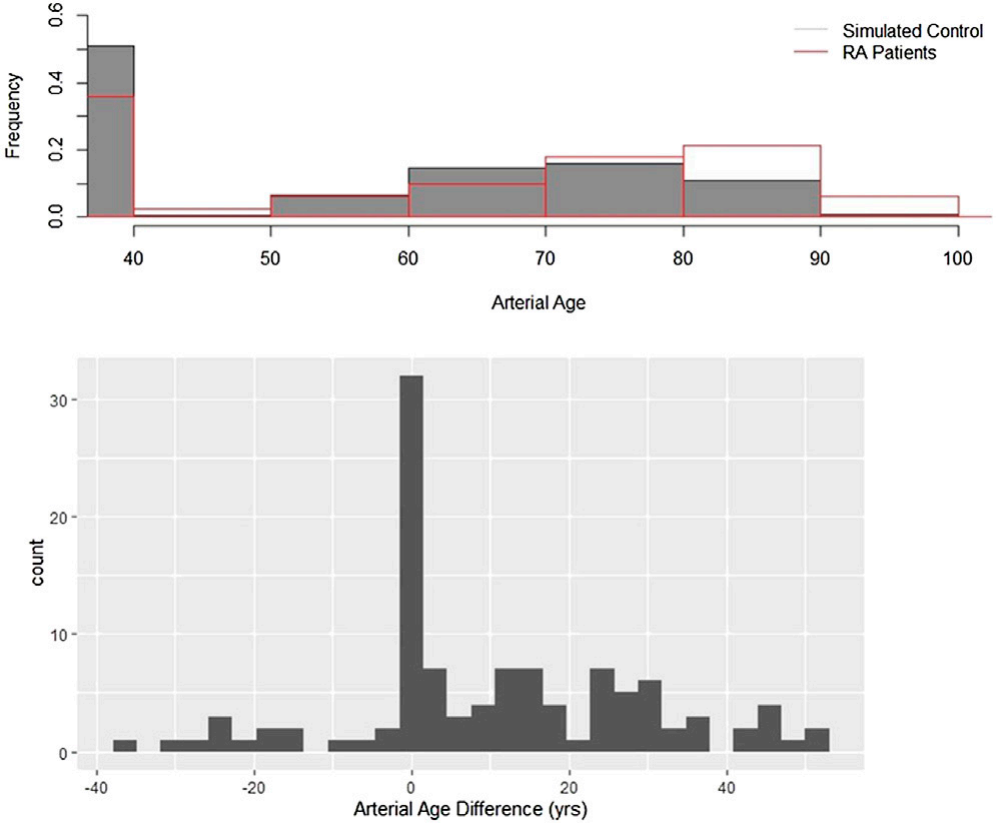
Arterial age in rheumatoid arthritis. Upper panel: Arterial age, converted from the observed and age-, gender-, and race-adjusted simulated coronary calcium scores. The figure shows the resulting distributions of the observed and simulated data. The shaded bars are the simulated control subjects, and the transparent bars with red contours represent the observed values. Lower panel: Arterial age distributions of patients with RA and the control populations are different. artAge_dif is the difference between the observed and the median of the control arterial ages. If there is no difference between RA patients and control subjects, then the histogram of artAge_dif must be symmetric around zero. Using Wilcoxon signed test, we showed that this null hypothesis is unlikely (*p* < 0.001).

CCS were measured in 112 RA patients; control CCS were obtained from the MESA database. Figure 1 upper panel compares the two arterial age distributions. Both histograms can be split into two parts. There is one single outstanding bar which represents subjects with zero CCS, and the arterial age of these subjects is by definition exactly 39.1. A characteristic feature of both curves a rightly skewed distorted bell-shape kind of part ranging from 39.1 up to hundred. These parts correspond to subjects having higher than zero CCS. Although the general features are similar, there are noteworthy differences between the two histograms. Smaller percentage of RA patients have zero CCS, and compared to controls, the center of the histogram is shifted to right, toward older ages. These visual impressions were confirmed by the statistical analysis. The CCS calculator gave the probability for each patient having higher than zero calcium. The average of these predicted probabilities was 0.517, while the found ratio is 0.642. The difference between the groups is highly significant (*p* = 0.008).

The assumption that arteries are older in RA compared to the matched controls was further tested with the help of variable artAge_dif, which is the difference of the observed value from the median of the controls. Statistical theory suggests that if there is no difference between the MESA and RA populations, then the distribution of artAge_dif has symmetric distribution around zero. Figure 1 lower panel shows that the difference distribution is not symmetric but clearly right skewed with a median of 6.34 years. This difference from the expected zero is highly significant (*z* = 5.51, *p* < 0.0001). Because artAge_dif has right skewed distribution (Figure 1 lower panel), the mean difference is higher than the median. As Table 2 shows, the mean difference is 10.45 (SD: 18.45) years, which means that the arteries of the RA patients in average are 10.45 years older than those of their MESA counterparts’ (*p* < 0.001).

### Correlation of Inflammatory Markers With Clinical Measures

As expected, the inflammatory markers (CRP and ESR) strongly correlated with each other and with the clinical disease activity (DAS28), data not shown. The correlation between artAge_dif and the disease duration is also significant (*r* = 0.22, *p* < 0.05).

### Comorbidities, Autoantibodies (Anti-Cyclic Citrullinated Peptide/Rheumatoid Factor), Smoking, and the Arterial Age

**FIGURE 2:**
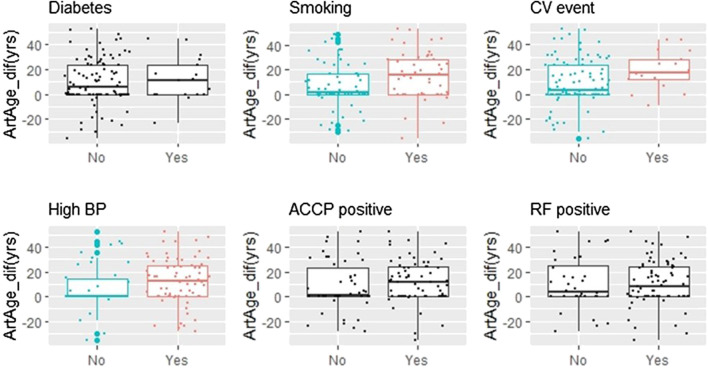
Smoking, CV events, and hypertension are associated with increased Artage_dif. Six binary variables were screened for potential effect on artAge_dif. The arterial age is increased significantly in patients who are smokers, have history of cardiovascular disease (CV event), or who are taking antihypertensive medicines (high BP). The corresponding *p* values (Kruskall–Wallis test): 0.016 (smoking), 0.029 (CV event), and 0.023 (high BP). Plots where the variable effects were significant are colored, while where the effect was not significant are in black (ACCP, anti-cyclic citrullinated peptides antibody; RF, rheumatoid factor).

Figure 2 displays the dependence of artAge_dif on categorical covariates. The differences between nonsmokers/smokers, patients without and with cardiovascular events, and patients with normal and high blood pressure were significant (*p* = 0.016, 0.029, and 0.023, respectively). By contrast, neither the presence of rheumatoid factor (RF), anti-cyclic citrullinated peptide (aCCP), nor diabetes proved to be predictive. We also checked if the previous statistical conclusions remain valid if we exclude diabetic patients’ data from the analysis. Seventeen patients (15.1%, Table 1) had diabetes. Excluding these individuals, 64.3% of the remaining patients had CCS above zero. This ratio is still significantly higher than the age- and gender-matched MESA control (49.4%, *p* = 0.004). The same is true for the difference from the matched medians (6.12 years, *p* < 0.001).

### The Effect of Cardiovascular Disease on the Coronary Calcium Score

Existing cardiovascular disease or history of cardiovascular events is associated with higher CCS (Lehker and Mukherjee, 2020). Subjects with existing cardiovascular condition were excluded from the MESA study, while 17 patients in our RA study group (15.18%, Table 1) had preexisting cardiovascular conditions. Therefore, the question arises to what extent the observed differences are due to the cardiovascular events. To answer this question, we split the RA study group into four subpopulations using the criteria that a patient had a zero CCS or the CCS was above it and that a patient had or had not previous cardiovascular disease. In the left panel of Supplementary Figure S1, proportions of patients with zero CCS are compared. Both RA subgroups are significantly different from their corresponding matched samples although the difference is much more pronounced in patients with previous cardiovascular disease (Supplementary Figure S1) (*p* = 0.023 and *p* = 0.005 respectively). Such difference between the two RA patient subgroups is not seen in the right panel of Supplementary Figure S1, and the medians of the matched arterial age differences are practically the same (16.7 and 18.1 years) for patients without and with cardiovascular disease. Both of them are significantly different from the expected zero (*p* < 0.001). These data suggest that the effect of RA on the CCS is mostly due to the accelerated progression rate. Cardiovascular disease is an additional risk factor because patients with cardiovascular disease have accelerated conversion rates from being CCS negative to CCS positive. The rate of conversion is significantly higher not only to control (*p* = 0.005) but also compared to RA patients without cardiovascular disease (*p* = 0.012).

### Arterial Aging Is More Accelerated in the First 10 Years of the Disease

**FIGURE 3:**
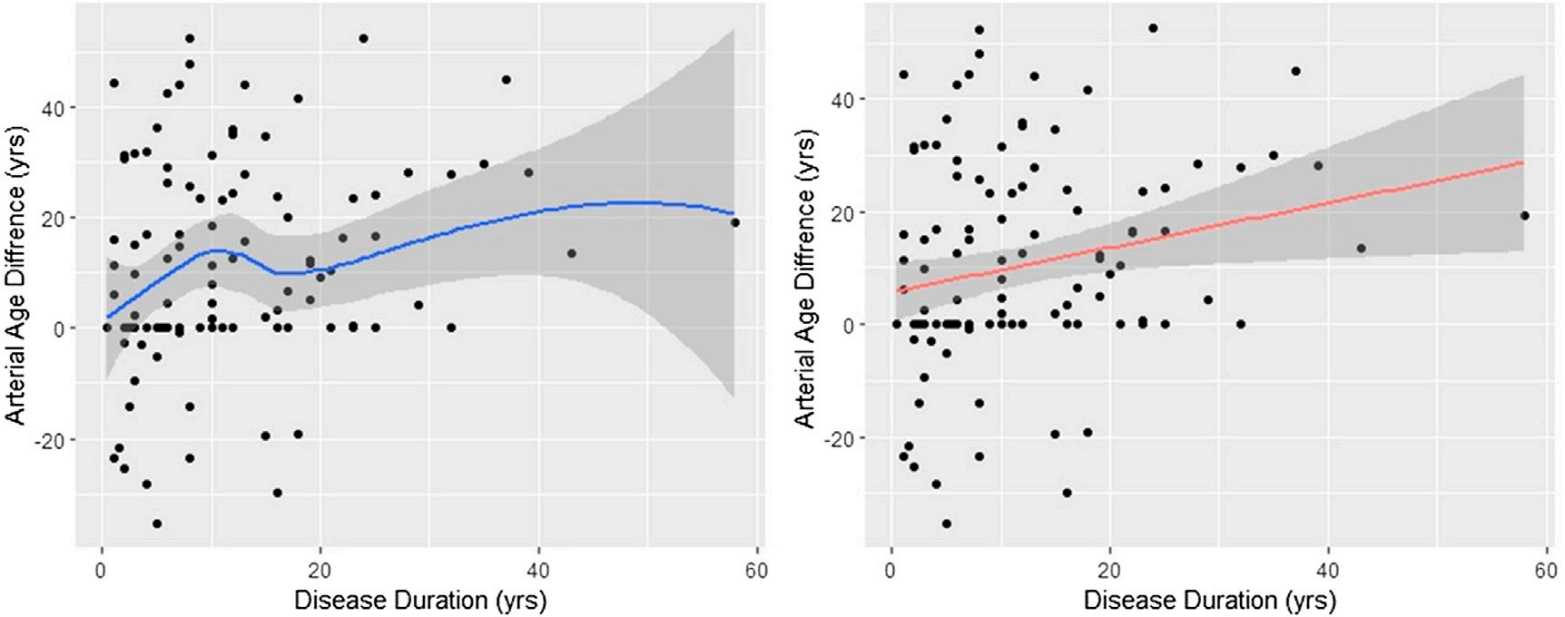
Arterial age difference depends on the disease duration. The effect of disease duration on the arterial age difference between RA patients and controls is shown. The smoothed curve in the left figure was obtained with Lowess. On the right figure, the red line represents the fitted regression line. The slope of the regression line (slope = 0.395, *t* = 2.74, *p* = 0.007) is the yearly divergence rate from the control. In both cases, the shaded areas around the lines are the 95% confidence intervals. The arterial aging is approximately 40% accelerated.

The analysis presented above suggests that the rate of the calcium build-up process in the control and RA populations is markedly different. Indeed, the time from the onset of the disease (disdur) was the only variable which showed significant correlation with the arterial age acceleration (data not shown). However, as the left panel of Figure 3 shows, the aging process is not constant. It increases monotonically (*r*_s_ = 0.249, *p* = 0.008), but the fitted nonparametric regression line (span = 0.9) suggests that the increase is faster in the first 10 years. Nevertheless, to get a numerical estimate of the arterial aging relative to the control, we fitted linear regression between disdur and artAge_dif. The fitted regression line is displayed in the right panel of Figure 3. The slope of the regression line is 0.395 (95% CI 0.10–0.68), significantly different from zero (*t* = 2.74, *p* = 0.007). The meaning of this 0.395 is that difference from the control increases by 0.395 years in every year of the disease; thereafter, in average, every year with RA contributes 0.395 extra arterial years.

### Progression Rate Estimation Using Follow-Up Data

**FIGURE 4:**
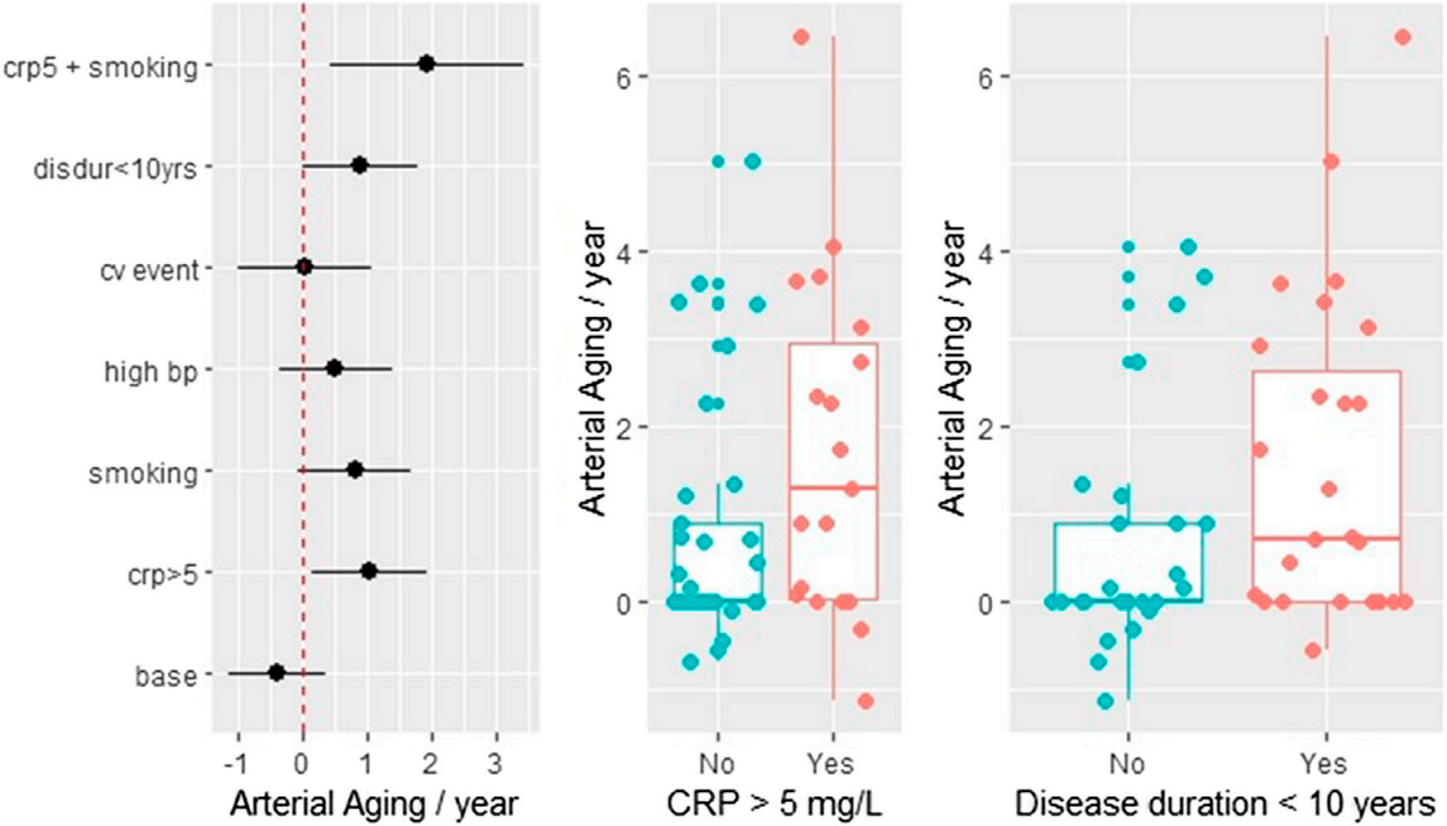
Follow-up data: the effect of inflammation, CV diseases, smoking, and RA disease duration on the arterial aging. The arterial aging rate was calculated by dividing the difference of the two consecutive arterial age estimates with the time interval between the two measurements. The left panel shows least-square estimates of five risk factors on the rate: smoking, disease duration ≤10 years (disdur < 10), history of cardiovascular disease (cvevent), CRP 5 > 5 mg/L, and concomitant antihypertensive drug taking (bp_high). When the 95% confidence intervals do not cross zero the effects are significant, which is true only for crp5 (*p* = 0.024) and disdur < 10 (*p* = 0.05). In the absence of any risk factors (which includes that the disease started more 10 years ago), the aging rate is close to zero (base). The combined effect of two risk factors such as CRP and smoking shown in the figure is an estimated marginal effect. The two boxplots illustrate the effect of crp5 and disdur < 10.

Fifty-four patients had a follow-up CCS measurement. Supplementary Table S1 summarizes the DAS, CRP, and ESR values at the time of the two measurements. Generally, there is strong correlation between these values (data not shown). The average time between the CCS scans was 1.28 ± 0.35 years. The annual arterial aging rate was calculated by dividing the difference between the second and first arterial age measurements with the length of the time interval between the two measurements. The least-square estimates with the corresponding 95% confidence intervals are shown in the left panel of Figure 4. The estimated aging rate is 1.44 (95% CI 0.77–2.11), which is in good agreement with the previous regression estimate. Nevertheless, from the further analysis, we excluded two subjects who converted during the observation period (i.e., their first CCS was zero and the second above zero, with estimated aging of 8.1 and 12.8 years, respectively), because from a statistical viewpoint, they were gross outliers. Without these two values, the rate estimate was 1.09 + 0.22, still highly different from zero (*p* < 0.001, *t* = 4.78). Additional regression analysis showed that the aging process is significantly faster in patients who had elevated CRP (>5 mg/L) level (*p* = 0.024) and in case the time since diagnosis is less than 10 years (*p* = 0.05). The least-square approach allowed us to investigate the combined effect of two or more risk factors. Figure 4 demonstrates that in smokers with elevated CRP levels, the arterial aging rate is doubled. The additionally computed nonparametric test confirmed the significance of the elevated CRP (*p* = 0.0494). The middle and the right panels of Figure 4 further demonstrate the effect of CRP and disease duration <10 years on the arterial aging.

### Effect of Medications on the Coronary Calcium Score

From the fifty-four patients in the follow-up study, thirty-one received biological therapy (twenty-seven patients anti-TNF-alpha, four patients non–anti–TNF-alpha bDMARDs) and twenty-three csDMARD therapy only. The yearly arterial aging progression rate in the follow-up group in patients receiving bDMARD therapy was numerically lower (1.16 ± 0.35) than that in patients receiving csDMARDs (1.73 ± 058); the difference (0.57 ± 0.67) was not significant (*t* = −0.85, *p* = 0.389).

## Discussion

Primarily due to cardiovascular comorbidities, patients with RA die significantly earlier (Nurmohamed et al., 2015; Nagy et al., 2018), and the risk of sudden cardiac death is doubled in RA compared to the general population (Maradit-Kremers et al., 2005; Naranjo et al., 2008; Karpouzas et al., 2014; Jagpal and Navarro-Millan, 2018). Due to the accelerated cardiovascular risk, the precise risk evaluation is essential. Our present data confirm and extend previous observations regarding the increased cardiovascular risk in RA. Here, we show for the first time the profound effect of RA on the arterial age, compared to the MESA population. Older arterial age was associated with smoking, previous cardiovascular events, and hypertension. The follow-up substudy was self-controlled and highlighted the importance of other additional factors. Ongoing inflammation (CRP > 5 mg/L), especially in smokers, and shorter disease duration (<10 years) accelerated arterial aging according to our follow-up data. Therefore, the increased cardiovascular risk due to RA increases with the disease years, but the augmentation is not linear, and in the first 10 years of the disease, the arterial aging is apparently more pronounced.

Inflammation plays a central role in the pathogenesis of atherosclerosis; elevated levels of C-reactive protein (CRP), interleukin-6, and N-terminal pro-hormone B-type natriuretic peptide (NTproBNP) correlate closely with cardiac events (Sabatine et al., 2004). Epidemiological studies suggested that chronic inflammation is associated with higher cardiovascular risk. In addition, inflammation is a recognized risk factor of AMI in RA (Meissner et al., 2016). A number of inflammatory mediators have been widely studied, both as surrogate biomarkers and as causal agents, in the pathophysiological network of atherogenesis and plaque vulnerability (Libby et al., 2002). Moreover, it was suggested that inflammatory processes and cytokines are similar in RA and in atherosclerotic vascular diseases (Plein et al., 2020). Low disease activity is associated with decreased risk of CVE in RA (Arts et al., 2017).

In patients with RA, inflammatory markers, disease severity, and RF positivity were found to be associated with the risk of atherosclerosis (Sattar et al., 2003). ACPA and RF are both unfavorable prognostic factors in RA; in accordance with our present data, both autoantibodies are independent of the accelerated arterial aging in RA (Berendsen et al., 2017).

The age is an independent risk factor for cardiovascular diseases; nevertheless, often the atherosclerotic disease burden is discordant with a patient’s chronological age. Calcium is a general component of the atherosclerotic plaque, but not that of the normal vessel wall (O’Rourke et al., 2000). Because of this structural difference, calcium is an accurate index of atherosclerotic disease burden and a useful tool to estimate the risk of cardiovascular adverse outcomes (Raggi et al., 2000; Kondos et al., 2003). Previous studies showed the importance of age-specific CCS percentiles to predict the occurrence of a cardiovascular event in patients with a similar risk profile (Wong et al., 2002). CCS is a widely accepted marker of coronary atherosclerosis. In the MESA population, a doubling of the CCS increased the probability of a coronary event by 25% in a 3.8-year follow-up period. Importantly, this predictive value was relatively stable across different ethnic groups (Detrano et al., 2008). Similar to our present data, RA severity was associated with the greater prevalence of coronary artery calcification than the MESA population (Giles et al., 2009). The Framingham risk score includes age, gender, total and HDL cholesterol, blood pressure, diabetes, and smoking. However, long-standing patients with RA had higher Framingham risk scores than patients with early disease or control subjects. Furthermore, long-standing inflammation represents additional cardiovascular risk (Chung et al., 2006). Moreover, the presence of CAC has been shown in early RA as well (Logstrup et al., 2017). The lack of diabetes effect in our study was somewhat surprising because it is generally presumed that the CCS is independently associated with incident coronary heart disease in diabetes (Malik et al., 2017). Framingham risk based on arterial age is more predictive of short-term incident coronary events than Framingham risk based on the observed age (McClelland et al., 2009).

According to recently published data, coronary artery calcification increases with higher total prednisone dose; by contrast, methotrexate and other csDMARDs do not influence coronary plaque progression (Karpouzas et al., 2020). Furthermore, DMARD and TNF-α antagonists are associated with reduced risk of myocardial infarction, stroke, and cardiovascular death (Micha et al., 2011; Westlake et al., 2011).

We could demonstrate neither positive nor negative effect of the applied drug therapy on the arterial aging process. It was somewhat surprising that anti-inflammatory drug therapy did not have a clear effect on the arterial aging process, in our present study. This could be explained by the relatively low number of patients and the limited period covered in the follow-up study. By contrast, in a recent study, another cardiovascular marker, vascular stiffness, significantly and consistently improved in RA patients, treated with biological or conventional synthetic DMARD therapy. RA has profound impact on the vasculature; already at time of the diagnosis, early RA patients have reduced vascular distensibility (Plein et al., 2020). Impaired endothelial function, a key event in the progression of atherosclerosis, was observed in RA. In addition to synovial lesions inflammation leads to vessel wall involvement as well. It was reported that endothelial dysfunction can be improved during anti–TNF-alpha therapy (Hürlimann et al., 2002). However, the limited study power prohibits to draw any definite conclusions regarding the effect of statins and targeted therapies on arterial aging. There are other limitations of our work as well: most of our patients had moderate disease activity, and untreated patients with early disease were not included in this study.

## Conclusion

RA significantly accelerates arterial aging; additionally to other risk factors, inflammation might be the pathophysiological link between RA and the increased calcification process. Further large-scale studies are needed to investigate the potential clinical benefit of CCS measurement in RA patients with risk factors for ischemic coronary heart disease.

## Data Availability Statement

The raw data supporting the conclusions of this article will be made available by the authors, without undue reservation.

## Ethics Statement

The studies involving human participants were reviewed and approved by both national and institutional ethics committees. IF 567-4-2016. The patients/participants provided their written informed consent to participate in this study.

## Author Contributions

NM performed design of the study, data collection, first draft of the manuscript, and data interpretation; ZT performed design of the study, data collection, and data interpretation; LT performed statistical analysis; AB, AS, and AIN performed data collection; DB and PMH performed data interpretation; and BM and GN performed design of the study, manuscript preparation, and data analysis. All authors have approved the submitted version. All authors have agreed both to be personally accountable for the author’s own contributions and to ensure that questions related to the accuracy or integrity of any part of the work, even ones in which the author was not personally involved, are appropriately investigated, resolved, and the resolution documented in the literature.

## Funding

This publication was supported by the National Research, Development, and Innovation Office of Hungary (NVKP_16-1-2016-0017 National Heart Program) and by Hungarian Scientific Research Fund Grant K 131479. The authors are grateful for the investigators, the staff, and the participants of the MESA study. AI Nagy was supported by the János Bolyai Scholarship of the Hungarian Academy of Sciences.Thematic Excellence Programme (2020-4.1.1.-TKP2020) of the Ministry for Innovation and Technology in Hungary, within the framework of the Therapeutic Development and Bioimaging programmes of the Semmelweis University.

## Conflict of Interest

The authors declare that the research was conducted in the absence of any commercial or financial relationships that could be construed as a potential conflict of interest.

## Glossary

aCCP: anti-cyclic citrullinated peptide
AMI: acute myocardial infarction
CAC: coronary artery calcium
CCS: coronary calcium scores
CI: confidence interval
CRP: C-reactive protein
CT: computed tomography
CV: cardiovascular
CVE: cardiovascular event
DAS28: disease activity score-28
DMARD: disease-modifying antirheumatic drugs
ECG: electrocardiography
ESR: erythrocyte sedimentation rate
HDL: high-density lipoprotein
IL-6: interleukin-6
LOWESS: locally weighted scatterplot smoothing
MESA: multi-ethnic study of atherosclerosis
NSAID: nonsteroidal anti-inflammatory drug
NTproBNP: N-terminal pro-hormone B-type natriuretic peptide
RA: rheumatoid arthritis
RF: rheumatoid factor
TNF: tumor necrosis factor
