# Supplementary material for: Largely Accelerated Arterial Aging in Rheumatoid Arthritis Is Associated With Inflammatory Activity and Smoking in the Early Stage of the Disease
Source: Front Pharmacol. 2020 Nov 26;11:523962. doi: 10.3389/fphar.2020.601344 (PMC7774279; doi:10.3389/fphar.2020.601344)
Supplement: Supplementary file 1 [file table1.docx]

| **Variable** | **N** | **Mean** | **SD** | **Min** | **Max** | **Median** |
| --- | --- | --- | --- | --- | --- | --- |
|  |  |  |  |  |  |  |
| **DAS** | 54 | 3.06 | 1.45 | 0.69 | 6.63 | 2.91 |
| **kDAS** | 50 | 2.96 | 1.39 | 0.69 | 6.60 | 2.8 |
| **CRP** | 54 | 7.91 | 15.08 | 0.16 | 79.00 | 3.6 |
| **kCRP** | 53 | 7.10 | 9.65 | 0.10 | 55.00 | 3.745 |
| **ESR** | 54 | 20.35 | 14.82 | 2.00 | 65.00 | 17.5 |
| **kESR** | 47 | 21.98 | 19.94 | 1.00 | 80.00 | 17 |
| **interval (yrs)** | 54 | 1.28 | 0.35 | 0.88 | 2.61 | 1.172603 |

**Supplementary Table 1. Follow-up data: summary of DAS and inflammatory markers** **measured at the time of first and second coronary calcium score measurements.**

Interval: time between the first and second CCS measurements.
