# Supplementary figures and images for: Largely Accelerated Arterial Aging in Rheumatoid Arthritis Is Associated With Inflammatory Activity and Smoking in the Early Stage of the Disease
Source: Front Pharmacol. 2020 Nov 26;11:523962. doi: 10.3389/fphar.2020.601344 (PMC7774279; doi:10.3389/fphar.2020.601344)

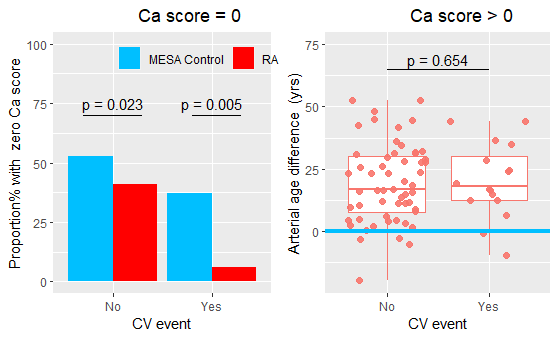

Supplement: Supplementary file 2 [file image1.tiff]
